# Profiling the immune tumor microenvironment of pediatric brain tumors with cavitron ultrasonic surgical aspirator (CUSA)-derived tissue fragments

**DOI:** 10.1093/noajnl/vdaf097

**Published:** 2025-05-17

**Authors:** Joyce I Meesters-Ensing, Mariëtte E G Kranendonk, Raoull Hoogendijk, Eelco Hoving, Friso G Calkoen, Jasper van der Lugt, Tiago Carvalheiro, Stefan Nierkens

**Affiliations:** Princess Máxima Center for Pediatric Oncology, Utrecht, the Netherlands; Princess Máxima Center for Pediatric Oncology, Utrecht, the Netherlands; Princess Máxima Center for Pediatric Oncology, Utrecht, the Netherlands; Princess Máxima Center for Pediatric Oncology, Utrecht, the Netherlands; Princess Máxima Center for Pediatric Oncology, Utrecht, the Netherlands; Princess Máxima Center for Pediatric Oncology, Utrecht, the Netherlands; Princess Máxima Center for Pediatric Oncology, Utrecht, the Netherlands; Center for Translational Immunology, University Medical Center Utrecht, Utrecht University, Utrecht, The Netherlands; Princess Máxima Center for Pediatric Oncology, Utrecht, the Netherlands

**Keywords:** pediatric brain tumor, cavitron ultrasonic surgical aspirator, tumor immune microenvironment, spectral flow cytometry, immunotherapy

## Abstract

**Background:**

Current treatment options for pediatric high-grade brain tumors are limited, with poor 5-year overall survival rates. While immunotherapy is promising for these patients, the composition of their tumor immune microenvironment (TIME) is still not fully understood, due to the limited availability of tumor material for research. Given the high abundance of tumor tissue fragments obtained using the cavitron ultrasonic surgical aspirator (CUSA), these samples could serve as a resource for research and diagnostic purposes.

**Methods:**

To evaluate CUSA tissue fragments as an alternative source for immune-landscape evaluation of brain tumors, we conducted immunological profiling on matched biopsy and CUSA-derived tissue fragments taken during resection from 11 pediatric brain tumor patients, using spectral flow cytometry and functional assays.

**Results:**

Cellular compositions were largely comparable between the two sources, both in freshly isolated and cryopreserved samples. Minor differences observed between biopsy and CUSA-derived tissue fragments from individual samples, likely reflect differences related with distinct tumor locations, caused by the small numbers of cells analyzed from one single biopsy versus multiple tumor sites collected with CUSA. Notably, expression of specific cellular immune subsets and their receptors indicating activation or regulation, were highly comparable between both materials, illustrating that CUSA can be used for detailed analyses of a multitude of immune cells and their functional markers. Moreover, CD8 + T-cells are enriched in tumor-infiltrating lymphocyte populations, maintaining their cytotoxic and proliferative capacity upon TCR (co)stimulation.

**Conclusions:**

Our findings demonstrate that CUSA-derived tissue fragments represent the TIME in pediatric brain tumors, offering a valuable sample resource for further research.

## Graphical Abstract

**Graphical Abstract:**
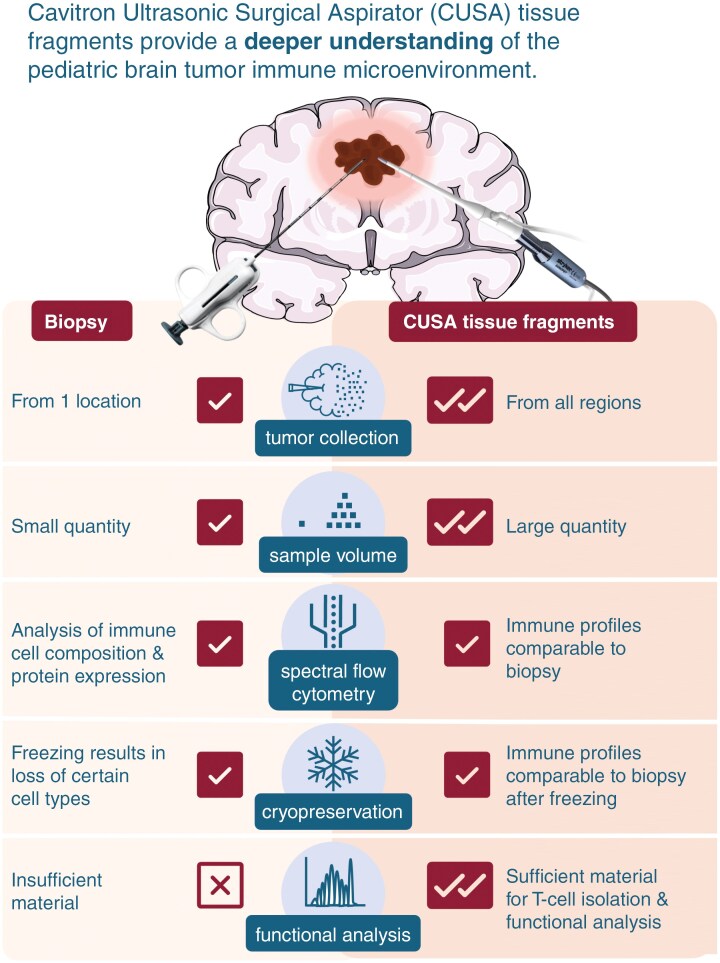

**Key Points**

- CUSA tissue fragments are suitable for immunological profiling of the TIME.
- T-cells isolated from CUSA tissue fragments retain their proliferative and cytotoxic capacity *ex vivo*.

**Importance of the Study**

Pediatric high-grade brain tumors have an overall poor survival rate and treatment options are limited. While immunotherapy offers promising new avenues, a deeper understanding of the tumor immune microenvironment (TIME) is essential to identifying possible therapeutic targets, increasing efficacy of the treatment. Traditional biopsy samples are generally limited in size and therefore pose a challenge in capturing brain tumor heterogeneity, therewith limiting information on TIME composition. This study demonstrates that CUSA-derived tissue fragments, often underutilized, represent an excellent alternative to biopsies for TIME analysis. Additionally, these fragments prove to be a valuable source of tumor-infiltrating T-cells that have retained their proliferative and cytotoxic capacity. Recognizing the potential of CUSA tissue fragments, this study could pave the way for more comprehensive tumor characterization and the development of targeted, effective immune treatments for pediatric brain tumor patients.

Brain tumors represent the most common solid tumors in pediatric oncology, constituting approximately 25% of all childhood cancers.^1^ Among these, the most prevalent high-grade brain tumors, classified as WHO grade 3 and 4, include diffuse pediatric-type high-grade glioma H3-wildtype and IDH-wildtype, diffuse midline glioma H3 K27-altered, diffuse hemispheric glioma H3 G34-mutant, medulloblastoma, ependymoma, and atypical teratoid/rhabdoid tumor. These high-grade brain tumors are particularly difficult to treat, contributing significantly to cancer-related childhood mortality. The 5-year overall survival rates for children suffering from high-grade brain tumors vary significantly between tumor types. While children diagnosed with medulloblastoma (MB) can achieve up to 70% long-term survival rates with current treatments, these numbers drop below 20% when patients relapse. In addition, diffuse midline glioma (DMG) is still considered incurable.^2–4^

Standard treatment protocols for pediatric brain tumor patients typically involve a combination of surgery, radiation, and chemotherapy, tailored to the specific tumor type.^5^ However, the efficacy of these treatments is often limited by various factors, like the feasibility of complete tumor resection, which depends on tumor location; radiation therapy that is not suitable for all age groups and generates serious long-term toxicity; and several chemotherapeutic agents do not effectively penetrate the blood–brain barrier.^5^ Given these challenges, there remains a significant unmet need for more effective therapeutic strategies. In this context, immunotherapy has emerged as a promising avenue, potentially offering new treatment options for these patients.

Over the past decades, immunotherapy has revolutionized the treatment of various cancers. Immune checkpoint blockade (ICB) has shown remarkable success in treating melanoma and other adult carcinomas,^6,7^ while chimeric antigen receptor T (CAR T) therapy has been effectively applied in both adult and pediatric hematological malignancies.^8,9^ However, the success of immunotherapy in pediatric solid tumors, particularly with ICB^10,11^ has been significantly limited. Dendritic cell vaccination and oncolytic viruses show promise in pediatric brain tumors, but with most clinical trials still in phase I and primarily focused on safety, it is still in early stages to conclude about their efficacy.^12^ To enhance the effectiveness of immunotherapy in brain tumors, a thorough understanding of the tumor immune microenvironment (TIME) is crucial. Although considerable knowledge has been gained from studies on adult brain tumors, these insights cannot be directly applied to pediatric cases due to distinct tumor types, such as embryonal tumors, the lower immunogenicity of pediatric tumors, and the more naive immune system in children.^13^

Recent studies on the TIME of pediatric brain tumors have revealed a diverse immune landscape, both between and within tumor subtypes. This heterogeneity is associated with various factors, including molecular subtypes and genetic alterations, but also with the tumor cell-type predominance.^14,15^ Whereas oligodendrocyte precursor cells within the tumor typically show an absence of immune activation signatures, astrocytes, and to a lesser extent mesenchymal-like cells, demonstrate significant enrichment for these pathways.^14^ This variability in immune profiles is further exemplified when comparing different pediatric brain tumor entities. For instance, the different medulloblastoma subtypes like infant SHH and SHH γ subtypes, exhibit higher levels of tumor-infiltrating B cells, while diffuse midline glioma (DMG) often presents a non-inflammatory microenvironment.^16^ This diversity complicates treatment strategies and underscores the need for more personalized therapies. Achieving this goal requires a deeper understanding of the specific composition of the TIME across different pediatric brain tumor subtypes.

A small tumor fragment obtained during surgery is generally considered the gold standard for brain tumor diagnosis.^17^ However, the limited size of this fragment, hereafter named biopsy, poses a challenge in capturing the heterogeneity of brain tumors.^18^ In addition, biopsy material is primarily reserved for diagnostic purposes, limiting its availability for research.^19^ In most brain tumor surgeries, however, the bulk of the tumor is resected using a cavitron ultra surgical aspirator (CUSA), with fragments collected, along with rinsing fluid and blood. This material, often highly abundant, yet occasionally regarded as surgical waste, has the potential to serve as a valuable resource for brain tumor research. Although underrepresented in literature, CUSA tissue fragments have been used for tumor diagnosis, such as histology and mass spectrometry, and for research purposes, as tumor cell culture and secretome studies.^19–23^ Yet, its use for studying the TIME and its function remain rare. Notably, Jacobs et al.^24^ demonstrated that the lymphocyte fraction, particularly regulatory T-cells (Tregs), in CUSA tissue fragments acquired from 83 adult brain tumor patients is comparable in frequency and function to those in biopsy material. These authors also showed that Tregs accumulate in high-grade brain tumors and can strongly suppress tumor-infiltrating T-cells.^24^

Here, we hypothesize that, unlike a single biopsy sample, CUSA tissue fragments can provide a more comprehensive representation of tumor heterogeneity, as it is collected from multiple tumor regions. To test whether CUSA tissue fragments can serve as an alternative to biopsies for investigating the TIME, we employed spectral flow cytometry to analyze both cell composition and protein expression profiles. In this study, we aimed to (1) compare the cellular composition and protein expression profiles of matched patient biopsy and CUSA tissue fragment samples, (2) investigate the impact of cryopreservation on cellular composition and protein expression profiles in both sample types, and (3) evaluate the suitability of CUSA-derived immune cells for performing functional assays. By addressing these objectives, we seek to validate CUSA tissue fragments as a reliable alternative to traditional biopsies for comprehensive TIME analysis, potentially enhancing our understanding of tumor heterogeneity and improving cancer research and treatment strategies.

## Materials and Methods

### Ethics Statement

Brain tumor material was collected from 19 patients treated at the Princes Máxima Center for pediatric oncology (Supplementary Table 1). Informed consent was given by all patients as participants of the overarching monitoring of the immune microenvironment (MIMIC) study (NTR NL8967). This study was approved by the MedNec medical ethical review committee to which the Princes Máxima Center is affiliated. Healthy adult donor blood was obtained after informed consent from blood donors at the University Medical Center Utrecht, Utrecht, The Netherlands.

### Sample Collection

Patient and healthy adult donor blood was collected in a standard EDTA tubes. Patient blood was drawn under anesthesia, prior to surgery. Tumor types included 11 WHO grade 4 tumors (medulloblastoma, *n* = 9 (group 3, *n* = 3; group 4, *n* = 2; not otherwise specified, *n* = 4); diffuse midline glioma H3.3 K27M, *n* = 1; diffuse high-grade glioma H3/IDH-WT, *n* = 1), 6 WHO grade 3 tumors (ependymoma, *n* = 6 (PFB, *n* = 1; PFA, *n* = 3; and ZFTA-RELA, *n* = 2)) and 2 WHO grade 1 tumors (pilocytic astrocytoma and Rosette-forming glioneuronal tumor). Where possible, matched tumor (biopsy) and CUSA tissue fragments were obtained from each patient during surgery. During surgery, biopsies were taken at the surgeon’s discretion, whereas the majority of the resection was performed using a standard cavitron ultra surgical aspirator (Integra®CUSA Excel). CUSA material was collected in a sterile bag, and biopsy material was stored in DMEM-F12 containing 10 mM Hepes, 1% Glutamax Supplement and 1% PenStrep until further processing on the same day.

Peripheral blood mononuclear cell (PBMC) isolation was performed according to local procedure by density gradient centrifugation using Ficoll-Paque Plus. Remaining red blood cells were removed by NH_4_Cl lysis and cells were frozen for long-term storage in liquid nitrogen.

### Tumor Handling and Dissociation

CUSA material was kept cold throughout the entire surgical procedure to prevent deterioration of the material. Both CUSA and biopsy material were processed within 1 h after collection from surgery.

A flow scheme of the tumor dissociation procedure can be found in Supplementary Figure 1. Briefly, CUSA material was filtered and collected tissue fragments were rinsed with PBS. Biopsy material was cut into small pieces if needed. Up to 1.5 g of materials was added to a gentleMACS^TM^ C-tube containing RPMI and 0.11 U/mL neutral protease (Nordmark, # 40459110).^25^ The gentleMACS tissue dissociator (Miltenyi, #130-093-235) was used for three rounds of dissociation with intermittent incubation of 30 min at 37°C. The material was then passed through a cell strainer and cells pelleted. Remaining red blood cells were removed by NH_4_Cl lysis. After washing, cells were resuspended in RPMI supplemented with 20% FCS, passed through a cell strainer and white blood cell (WBC) content estimated using the Sysmex XN-350 cell analyzer. Part of the cell suspensions was stored O/N at 4°C for flow cytometric analysis and T-cell proliferation assay. Remaining material was cryopreserved and stored in N_2_ at cell concentrations of at least 10 × 10^6^ WBCs/mL.

### Flow Cytometry Analysis

Fresh or freshly thawed cells were analyzed with both myeloid and T/B/NK antibody-panels (Supplementary Tables 2 and 3). Samples were washed once with PBS and stained for 30 min at 4°C with ViaDye Red fixable viability dye (Cytek, #SKU R7-60008) in PBS, followed by a 30 min incubation at 4°C with extracellular marker antibodies. An unstained control was included. Cells were washed twice after each step with FACS-buffer (PBS/1%FCS). For the T/B/NK panel, cells were subsequently fixed for 30 min at 4°C using the eBioscience FoxP3 transcription factor staining buffer set (Thermo Fisher, #00-5523-00) and incubated for another 30 min at 4°C with intracellular marker antibodies. Cells were washed twice after each step with 1× Permeabilization buffer. Cells were resuspended in FACS-buffer and acquired on a Cytek Aurora spectral flow cytometer (5-laser 16UV-16V-14B-10YG-8R).

### Unmixing

Measurement and subsequent unmixing of samples were performed using SpectroFlo software (version 3.3.0) on Aurora (Cytek). For unmixing, single stains on both cells and Cytek® FSP™ CompBeads (Cytek, # SKU B7-10011) were prepared using the identical antibodies used for staining with the 2 panels. Spectra were saved to the instrument library. Additionally, the built-in module for extracting multiple autofluorescence signals was used to identify unique autofluorescence spectra in the unstained samples of CUSA tissue fragment and biopsy cell suspensions. The unique autofluorescence spectra together with the appropriate single stain reference controls were then used for unmixing. Dependent on the brightness of a marker in the fully stained sample, a choice was made to either include beads or cells as reference controls.^26,27^ Following correct unmixing, data files were exported for further analysis. Complete gating strategies for both Myeloid and T/B/NK-antibody panels can be found in Supplementary Figures 2 and 3.

### T-cell Isolation and Proliferation Assay

T-cells were isolated from fresh CUSA tissue fragments and healthy adult donor (HD) PBMCs via positive selection using CD3 human microbeads (Miltenyi, #130-050-101), according to the manufacturer’s instructions. Briefly, HD PBMCs or cells from a CUSA tissue fragment suspension were incubated with CD3 microbeads, after which labeled cells were separated from the bulk using a MS column (Miltenyi, #130-042-201), placed in a magnetic miniMACS separator (Miltenyi, #130-090-312). CD3^+^ T-cells were washed several times on the column before being eluted into a new tube. For extra purity of T-cells, the loading, washing and eluting of samples was performed twice using separate columns. After the second elution, T-cells were spun down and resuspended in PBS at a concentration of 2 × 10^6^ cells/mL. Cell trace violet (CTV; Thermo Fisher, # C34571) was diluted to 2 µM in PBS and equal volumes of cells and CTV were mixed thoroughly. Following a 10-min incubation at 37°C, culture medium (RPMI/5% human serum/1% PS) was added to stop the reaction. T-cells were washed and resuspended in culture medium at a concentration of 1 × 10^6^ cells/mL and 5 × 10^4^ plated in a 96-well U-bottom culture plate (Sarstedt, #83.3925). Human T-Activator Dynabeads™ (ThermoFisher, # 11131D) were added in a 1:25 bead:T-cell ratio and cells were incubated for 3 days at 37°C, 5% CO_2_. Supernatant was collected and T-cell proliferation was then assessed by flow cytometry. Cells were spun down, stained with extracellular markers for CD3-BUV396, CD4-BUV805, CD8-APC/Fire810, and CD45-cFluorV547 as described above. Sample acquisition and unmixing was performed on Aurora. Gating was performed in FlowJo and analysis was performed using the proliferation module of the software.

### Cytokine Analysis

A Legendplex™ human CD8/NK-panel (Biolegend, #741187) was used according to manufacturer’s instructions, to determine IL-10, TNF-α, IFN-γ, Granzyme B, and Granulysin concentrations in proliferation supernatant samples. Supernatant and standard control samples were incubated together with pre-defined beads for 2 h at room temperature under continuous shaking at 800 rpm. Beads were spun down and washed twice with washing buffer. Beads were then incubated with detection antibodies for 1 hour at room temperature and 800 rpm, followed by a 30-minute incubation with SA-PE. Beads were washed twice, resuspended in washing buffer and measured on Aurora. Exported fcs files were subsequently analyzed using LEGENDplex™ Data Analysis Software.

### Data Analysis and Statistics

Flow cytometry analysis for specific cell populations was carried out using FlowJo (v10.10.0) software. Further statistical analysis was performed using Graphpad Prism (v10.2.2). Wilcoxon tests were used to determine statistical differences between 2 paired sample groups, while a mixed effect model with Geisser Greenhouse correction was used for comparing multiple matched sample groups.

## Results

### Comparison of Immune Cell Infiltrate in Biopsy and CUSA Tissue Fragments from Pediatric Brain Tumors

**Figure 1.**
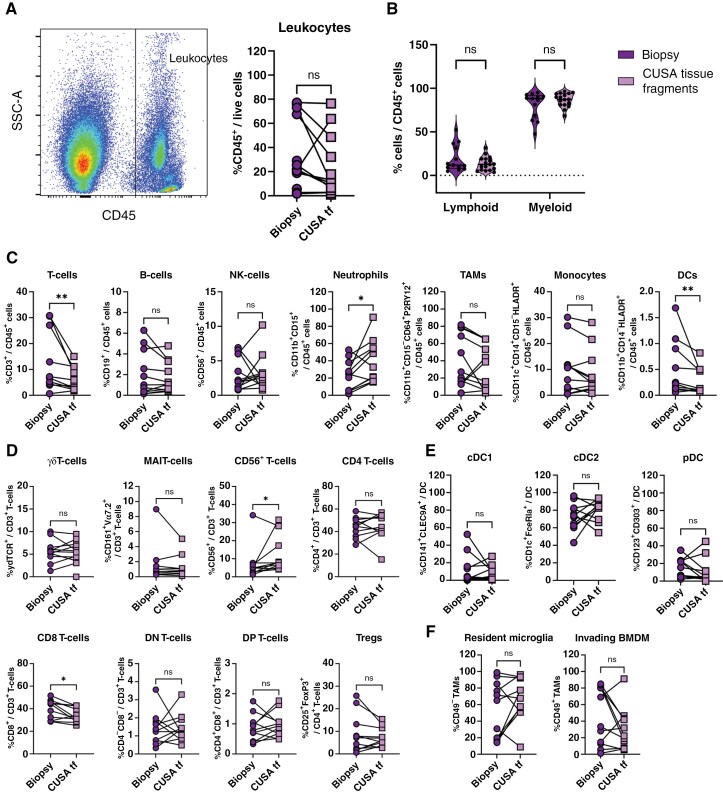
**Cellular composition of the TME in pediatric brain tumors is comparable between CUSA tissue fragments and biopsy material. (A) I**mmune cell infiltrate (CD45+) identified using spectral flow cytometry. (**B)** Comparison of the lymphocyte and myeloid compartment of biopsy and CUSA tissue fragment (tf) materials. (**C–F)** Comparison of the cellular composition of lymphocyte and myeloid subpopulations **(C)**, T-cell subsets **(D)**, DC subsets **(E)** and the different TAM subsets (F) in both types of samples. TAM = tumor-associated macrophage; cDC = conventional dendritic cell; pDC = plasmacytoid dendritic cell; MAIT = mucosal-associated invariant T-cell; DN = double negative; DP = double positive; Treg = regulatory T-cell; BMDM = bone marrow derived macrophage. Statistical differences in a Wilcoxon paired t-test are depicted as ns = non-significant, * = *P* < .05 and ** = *P* < .01; *n* = 11.

We first compared the immune cell infiltrate in freshly processed, matched biopsy and CUSA tissue fragments from 11 pediatric brain tumors using an optimized cell isolation protocol for brain tissue and spectral flow cytometry. The average numbers of total cells available for analyses were 2.5 × 10^7^ (0.4-3.9 × 10^7^) for biopsies and 7.1 × 10^8^ (5–10.7 × 10^8^) for CUSA tissue fragments. The analysis revealed an immune cell infiltrate (CD45^+^) ranging from 0.5% to 73% of total cells in both material types (Figure 1A). Despite the differences in the percentages of CD45^+^ cells, these were not statistically significant between biopsy and CUSA tissue fragments. Additionally, comparison of total CD45^+^ lymphoid (CD3^+^CD19^+^CD56^+^ SSC^low^) versus myeloid compartments did not show significant differences between the two sources (Figure 1B). However, a detailed analysis of the CD45^+^ populations, revealed significant differences in the frequencies of T-cells, neutrophils and dendritic cells (DCs) between materials. Specifically, CUSA tissue fragments exhibited increased neutrophil percentages and decreased DC and T-cell frequencies in CUSA samples compared to matched biopsy materials (Figure 1C). Biopsy samples with the highest T-cell content were investigated using immunohistochemistry and showed a heterogeneous spatial distribution of tumor-infiltrating T-cells within the tumor (Supplementary Figure 4). To further assess the relationship between cell populations in biopsy and CUSA tissue fragments, we performed a correlation analysis of the different lymphoid and myeloid subpopulations. Here, a moderate correlation was observed between biopsy and CUSA tissue fragments for all populations except NK-cells ( Supplementary Figure 5A and B).

To investigate whether the differences in DC and T-cell populations were skewed by the higher neutrophil numbers in CUSA tissue fragments, a normalization step was performed excluding neutrophils from the analysis. Post-normalization, no significant differences in DC or T-cell frequencies were found, although a trend toward lower T-cell content in the CUSA tissue fragments persisted (Supplementary Figure 6A). Further detailed analyses revealed that only the CD3^+^CD8^+^ population was increased in biopsy material, while the CD3^+^CD56^+^ population was higher in CUSA tissue fragments (Figure 1D). No additional differences were observed when comparing subpopulations of DCs (Figure 1E) or tumor-associated macrophages (TAMs) (Figure 1F). These findings highlight the feasibility of using CUSA material to investigate immune cell subset composition in pediatric brain tumors as an alternative to biopsies, with the advantage of CUSA providing a larger number of cells available for analysis.

### Protein Expression Profiles Are Comparable Between Biopsy and CUSA Tissue Fragments

**Figure 2.**
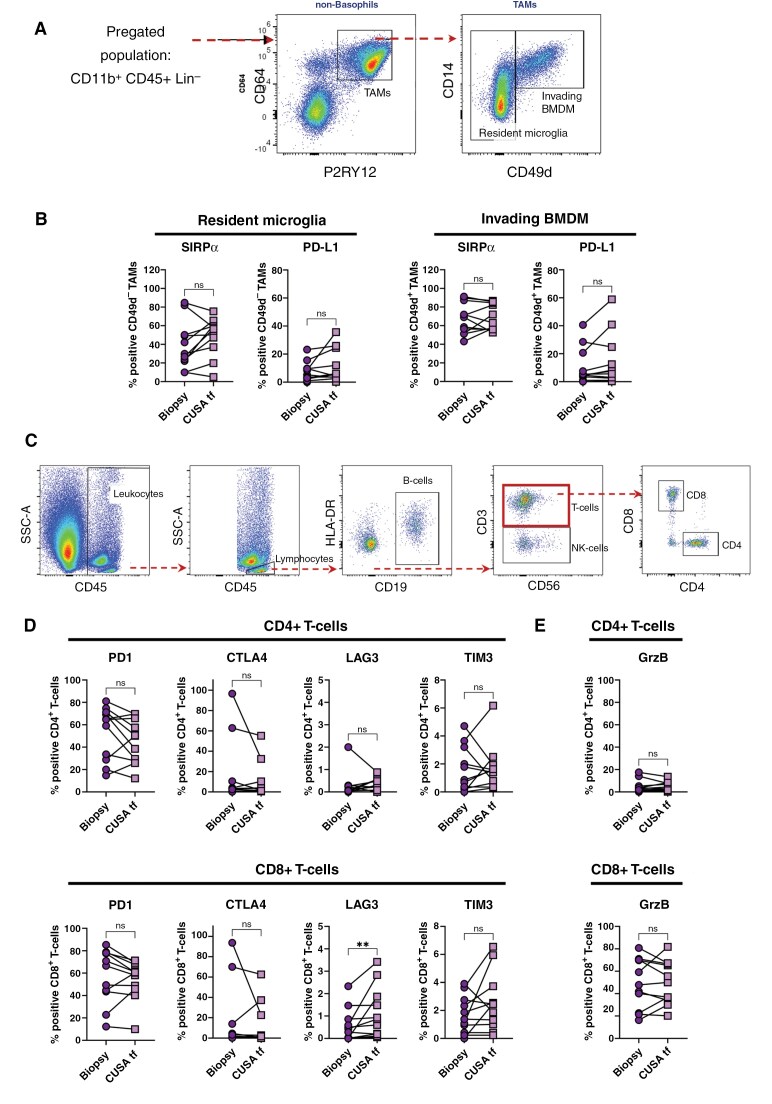
**Protein expression profiles of TAM- and T-cell subpopulations are comparable between biopsy and CUSA tissue fragment material. (A)** Flow cytometry gating strategy to identify brain resident microglia (Lin^-^CD11b^+^CD45^+^CD64^+^P2RY12^+^CD49d^-^) and invading BMDM (Lin^-^CD11b^+^CD45^+^CD64^+^ P2RY12^+^CD49d^+^). (**B)** Protein expression of SIRPα and PD-L1 on both resident microglia and invading BMDM in the different freshly isolated samples. (**C)** Flow cytometry gating strategy used to identify CD4^+^ and CD8^+^ T-cell subsets. (**D)** CD4^+^/CD8^+^ T-cell ratio of each matched patient sample. (**E)** PD1, CTLA4, LAG3, and TIM3 checkpoint receptor expression levels on CD4^+^ and CD8^+^ T-cells in the matched patient samples. (**F)** Granzyme B (GrzB) production on both CD4^+^ and CD8^+^ T-cells. Lin = lineage markers (CD3/CD19/CD56); TAM = tumor-associated macrophage; BMDM = bone marrow-derived macrophage. Statistical differences in a Wilcoxon paired t-test are depicted as ns = non-significant and ** = *P* < .01; *n* = 11.

Next, we compared the expression of markers for additional cell subsets and/or their maturation/activation status between freshly isolated biopsy and CUSA tissue fragment material. We focused on TAM and T-cell subsets (Supplementary Figures 2 and 3), as these are the most abundant in myeloid or lymphoid populations, respectively. TAMs in the TIME can be divided into brain resident microglia (CD49d^-^) and invading bone marrow-derived macrophages (BMDM, CD49d^+^) (Figure 2A and Supplementary Figure 2).^28^ The two TAM subsets were analyzed for their expression of Signal regulatory protein alpha (SIRPα/CD172a) and programmed cell death ligand 1 (PD-L1), two immune checkpoint markers expressed by macrophages. We observed that the expression levels of SIRPα and PD-L1 did not differ between biopsy and CUSA tissue fragment materials for neither of the TAM subsets (Figure 2B).

Tumor-infiltrating T-cells isolated from both biopsy and CUSA tissue fragment materials were divided into helper (CD4^+^) and cytotoxic (CD8^+^) T-cells (Figure 2C) showing similar expression levels of PD-1, CTLA-4, and TIM-3 checkpoint receptors between biopsy and CUSA tissue fragments. CD8^+^ T-cells isolated from CUSA tissue fragments, however, exhibited an increased expression of LAG3, when compared with biopsy (Figure 2D). Interestingly, CD8^+^ T-cells in the TIME were still able to produce Granzyme B, indicating that these cells have retained their cytotoxic capacity (Figure 2E). These findings suggest that CUSA tissue fragments can reliably reflect the cell subset populations and activation status in pediatric brain tumors, similar to biopsies.

### Cryopreservation Induces Minor Changes in Protein Expression in both Biopsy and CUSA Tissue Fragments

**Figure 3.**
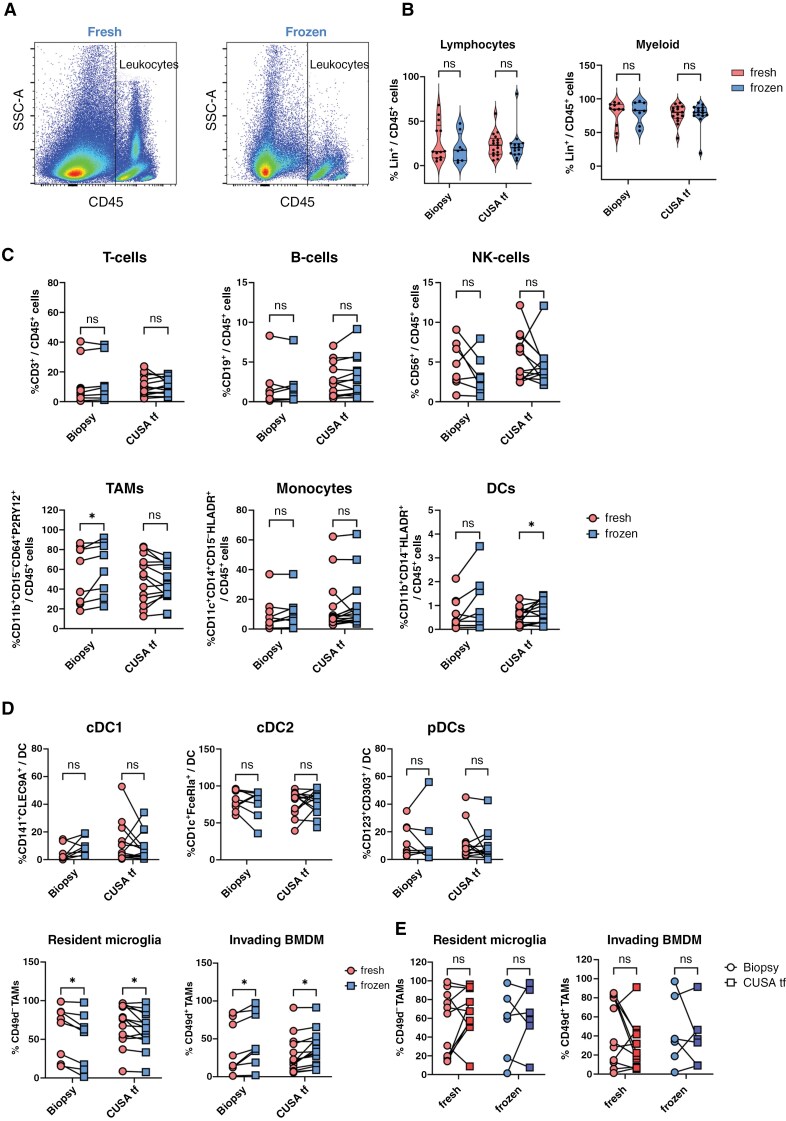
**Comparison of the immune cellular composition of the TME between freshly isolated and cryopreserved samples. (A)** Representative flow cytometry CD45 + dot plot showing the loss of granulocytes after cryopreservation. (**B–D)** Comparison of the cellular composition of lymphocyte and myeloid compartments in freshly isolated and cryopreserved biopsy and CUSA tf material **(B)**, lymphocyte and myeloid subpopulations **(C)** and DC and TAM subsets **(D)** after excluding granulocytes; *n* = 8 Biopsy, *n* = 15 CUSA tf. (**E)** Comparison of TAM subsets in biopsy and CUSA tf material in both freshly isolated and cryopreserved samples; *n* = 11 fresh, *n* = 6 frozen. TAM = tumor-associated macrophage; cDC = conventional dendritic cell; pDC = plasmacytoid dendritic cell; BMDM = bone marrow-derived macrophage. Statistical differences using multiple Wilcoxon paired *t*-tests are depicted as ns = non-significant and * = *P* < .05.

Since freshly isolated material is not always available, we investigated whether cryopreserved material could serve as an alternative. Polymorphonuclear cells, such as neutrophils, are highly sensitive to temperature fluctuations and are largely lost during a freeze-thaw-cycle.^29^ In line, we observed the same for neutrophils in both biopsy and CUSA tissue fragments (Figure 3A, Supplementary Figure 6B and C). This loss of neutrophils impairs direct comparisons of the cellular composition between freshly isolated and cryopreserved materials. Therefore, we normalized the data by excluding the neutrophils from the CD45^+^ population in both sample types. Within this normalized CD45^+^ population, cryopreservation did not significantly alter the overall composition of lymphocyte and myeloid (sub)populations in either material source (Figure 3B and C). To further support these findings, we correlated the percentages of recovered lymphocyte and myeloid subpopulations from matched cryopreserved and freshly isolated materials, confirming a strong correlation for all cellular subsets between both sample types, except for NK-cells ( Supplementary Figure 6D). Interestingly, the TAM and DC populations showed to be affected by cryopreservation in either biopsy or CUSA tissue fragments, although this effect could not be confirmed when examining the individual TAM and DC subpopulations (Figure 3D). No significant differences were observed for the DC subpopulations, while both biopsy and CUSA tissue fragments showed reduced frequencies of resident microglia and increased percentages of invading BMDM after cryopreservation. However, since these changes were consistent across both materials, they balance when comparing between either fresh or cryopreserved samples (Figure 3E). Consequently, data analysis yields comparable results when consistently comparing either fresh or cryopreserved biopsy and CUSA tissue fragments.

**Figure 4.**
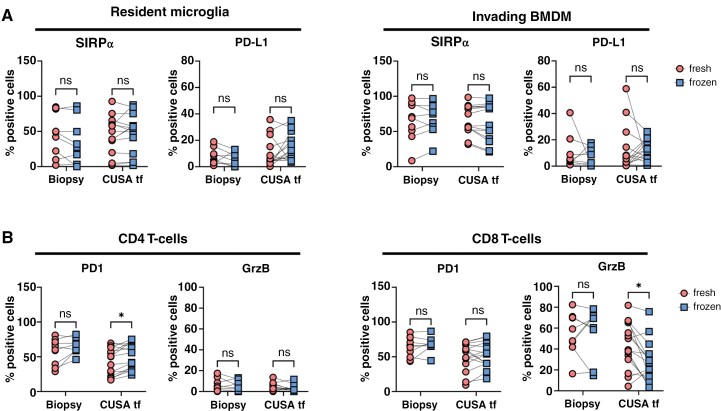
**Protein expression profiles on TAM- and T-cell subsets before and after cryopreservation. (A)** Protein expression levels of SIRPα and PD-L1 on TAM subsets upon cryopreservation. (**B)** PD1 and GrzB expression in both CD4^+^ and CD8^+^ T-cell populations upon cryopreservation. BMDM = bone marrow derived macrophage; GrzB = Granzyme B. Statistical differences using multiple Wilcoxon paired *t*-tests are depicted as ns = non-significant and * = *P* < .05; *n* = 8; Biopsy, *n* = 15 CUSA tf.

In addition to potential changes in cellular composition, we investigated the effect of cryopreservation on the expression of receptors and cytotoxic molecules related to the function of the different TAM and T-cell subsets. After cryopreservation, PD1 expression increased on CD4^+^ T-cells, while the percentage of CD8 + T-cells expressing Granzyme B decreased, with both differences observed exclusively in CUSA tissue fragments. Although similar trends were observed for both subsets across both materials, these differences were not statistically significant (Figure 4B, Supplementary Figure 7B). In addition, we found that cryopreserved CUSA tissue fragments exhibited higher SIRPα expression on resident microglia compared to cryopreserved tumor biopsy. Similarly, PDL-1 expression on invading BMDM was increased on CUSA tissue fragments compared to tumor biopsy (Figure 4A, Supplementary Figure 7A). Notably, when comparing SIRPα and PDL-1 expression on these TAMs subsets between tumor biopsy and CUSA tumor fragments from the same patients, either in fresh or cryopreserved samples, similar trends were observed. This suggests that the observed differences are more likely due to sample type or patient-specific factors rather than an effect of cryopreservation (data not shown).

In summary, cryopreserved material can be used as a substitute for freshly isolated material in studies comparing the cellular composition and protein expression in biopsy and CUSA tissue fragments, provided that proper normalization steps are applied. However, mixing cryopreserved and fresh samples within the same analysis should be avoided.

### Leukocyte Composition and T-cell Expression Profiles Are Unique to the TIME

The CUSA-derived samples consist of a liquid fraction, blood, and rinsing fluids collected during surgery, and tissue fragments, which we processed separately ( Supplementary Figure 1). To examine the cellular composition between the liquid fraction and tissue fragments, and to assess TIME signature representation in peripheral blood, we analyzed the cellular composition and protein expression profiles in the TIME compared to matched peripheral blood and “liquid” CUSA samples.

**Figure 5.**
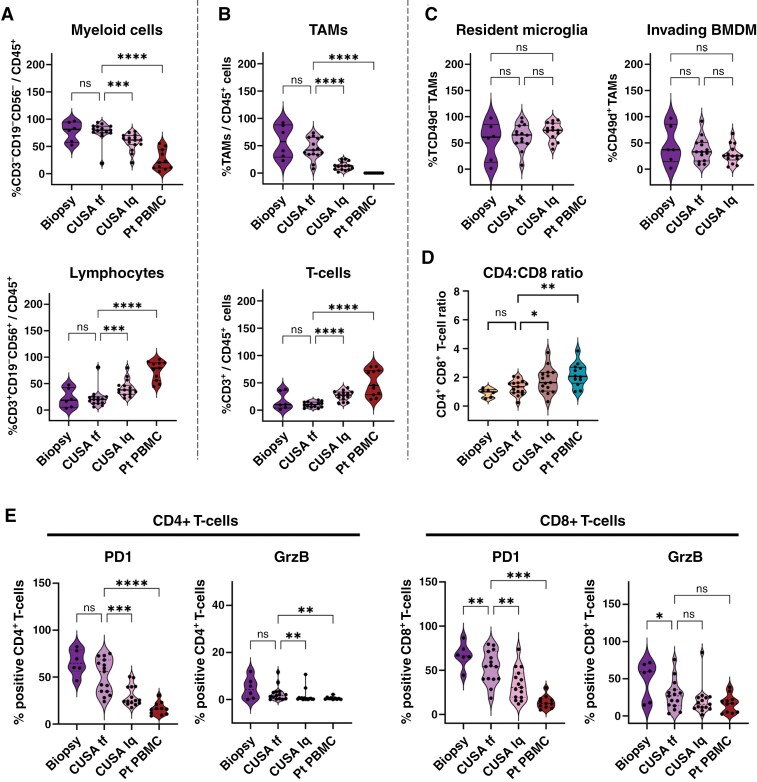
**The Immune cell composition and expression profiles of the TME in biopsy and CUSA tissue fragment (tf) are significantly different from the patient (Pt) peripheral blood.** Cryopreserved biopsy, CUSA tumor fragments, CUSA liquid (lq) fraction and peripheral blood (PBMCs) from pediatric brain tumors were analyzed for their (**A)** Myeloid and Lymphoid composition and (**B)** both TAMs and T-cell compartments. (**C)** Comparison of the cellular composition of TAM subsets and (**D)** CD4^+^/CD8^+^ T-cell ratios. (**E)** Protein expression levels of both PD1 and GrzB on CD4^+^ and CD8^+^ T-cell subsets. TAM = tumor-associated macrophage; BMDM = bone marrow-derived macrophage; GrzB = Granzyme B. Statistical differences using a mixed effect model with Greisser-Greenhouse correction and only matched samples are depicted as ns = non-significant, * = *P* < .05 and ** = *P* < .01 *** = *P* < .001 and **** = *P* < .0001; *n* = 6 Biopsy, *n* = 15 CUSA tf, *n* = 15 CUSA lq, *n* = 11 Pt PBMC.

Compared to PBMCs from patients, the leukocyte composition in the TIME (both biopsy and CUSA tissue fragments) was dominated by myeloid cells (Figures 1B, 3B, and 5A). Macrophages, which are typically absent in the blood, were the largest contributors to the myeloid population in the brain TIME (Figure 5B, upper panel). A lower frequency of TAMs was observed in the CUSA liquid fraction compared to the tumor fragments samples. However, despite the lower TAM frequency, the CUSA-derived liquid fraction showed similar frequencies of TAM subpopulations compared to the tumor fragments (Figure 5C).

In contrast, T-cells were significantly lower represented in the TIME compared to the CUSA liquid fraction and peripheral blood. The CD4^+^/CD8^+^ T-cell ratio was also lower in the TIME compared to both the CUSA-derived liquid fraction and peripheral blood from the same patients (Figure 5B, lower panel and 5D). These findings suggest that while T-cell numbers in the brain TIME are low, it is mostly infiltrated by cytotoxic T-cells. We also compared PD1 and Granzyme B expression levels on CD4^+^ and CD8^+^ T-cell subsets between peripheral blood and the TIME. While PD1 expression was significantly increased on T-cells from the brain TIME compared to peripheral blood, no differences were observed for expression of CTLA4, LAG3 and TIM3 (Figure 5E, Supplementary Figure 8A). Granzyme B expression was elevated in CD8^+^ T-cells from the TIME compared to peripheral blood, suggesting that these cells retained their functional capacity (Figure 5E, Supplementary Figure 8B). Thus, these findings indicate that, despite the low T-cell infiltration in the TIME, these cells seem to retain, at least partial cytotoxic capacity.

### Tumor-infiltrating T-cells Retain Their Proliferative Capacity

**Figure 6.**
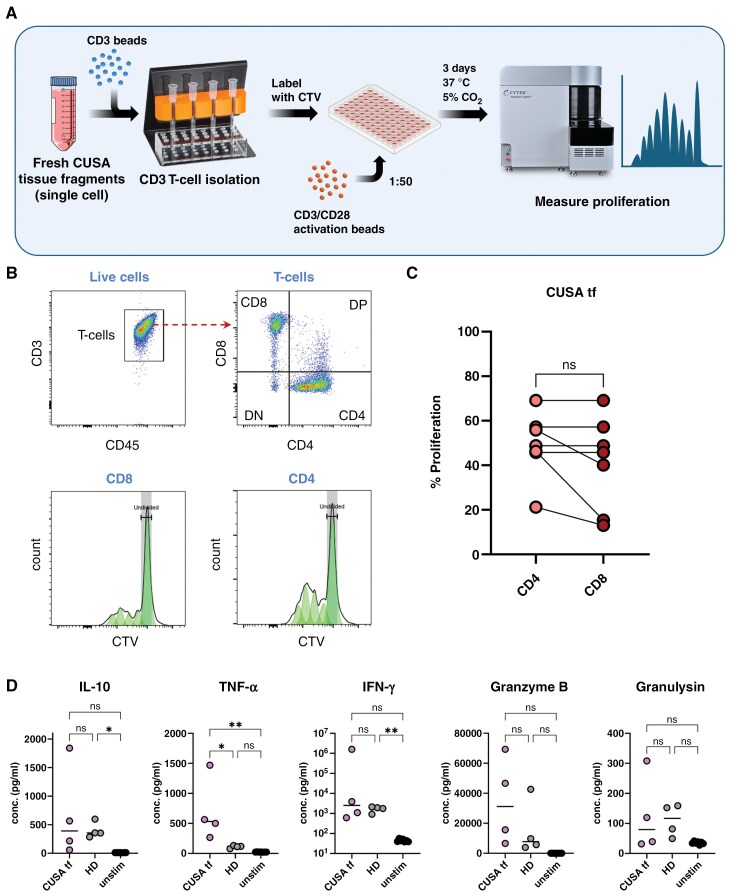
**T-cells isolated from CUSA-derived tissue fragment (tf) material are able to proliferate and produce pro-inflammatory cytokines. (A)** T-cell isolation and proliferation workflow using flow cytometry. Some graphical elements in this panel were adapted from Servier Medical Art (smart.servier.com), licensed under CC BY 4.0. (**B)** Representative flow cytometry analysis showing proliferation of CD4^+^ and CD8^+^ T-cells after 3 days of stimulation with anti-CD3/28 activating beads. (**C)** Percentage of divided CD4^+^ and CD8^+^ T-cells isolated from CUSA tissue fragments determined using the proliferation modeling tool of FlowJo; *n* = 7. (**D)** Production of pro-inflammatory cytokines IL-10, TNF-α, and IFN-γ and cytotoxic molecules Granzyme B and Granulysin by isolated T-cells after 3 days of stimulation with anti-CD3/28 activating bead, determined by Legendplex (Biolegend). Culture supernatant of adult healthy donor (HD) T-cells and non-stimulated (unstim) T-cells were used as a reference; *n* = 4.

To evaluate the functionality of tumor-infiltrating T-cells isolated from the TIME, we performed a T-cell proliferation assay (Figure 6A). T-cell isolation could not be performed from biopsies due to their limited sample size. In contrast, the large sample amount of CUSA-derived tissue fragment material allowed for successful T-cell isolation and subsequent T-cell proliferation assay. Interestingly, when removed from the suppressive environment of the TIME,^30^ T-cells were able to proliferate in response to CD3/CD28 polyclonal stimulation (Figure 6B). No significant difference in the proliferative capacity was observed between CD4^+^ and CD8^+^ T-cell subpopulations (Figure 6C). The functional potential of these tumor-infiltrating T-cells was further supported by the analysis of the pro-inflammatory cytokines and cytotoxic molecules secreted by TIME-derived T-cells. No significant differences were observed for the levels of IL-10, IFN-γ, and Granulysin, whereas the levels of TNF-α and Granzyme B were increased in the supernatants of tumor-infiltrating T-cells, when compared with HD control T-cells (Figure 6D). These data underline that the tumor-infiltrating T-cells are functional and could potentially be activated.

## Discussion

The ability to effectively treat children suffering from high-grade brain tumors using immunotherapy, requires a thorough understanding of the TIME. Historically, studies on the immune landscape of brain tumors have relied heavily on biopsy material.^31,32^ However, the limiting availability of biopsy samples poses challenges for in depth immune profiling, especially given that these samples may not adequately represent the heterogeneity of the tumor. Tumor cellular composition can vary significantly depending on the location of the biopsy, leading to biased or incomplete characterization.^33,34^ In light of these limitations, this study explores the use of tissue fragments obtained from CUSA as an alternative source for immune profiling, as it is collected from all regions of the tumor.

Our data show that CUSA tissue fragments could serve as a valuable substitute for biopsy material and may even be a better representation of the TIME, since a larger number of cells can be analyzed. In addition, we employed spectral flow cytometry, as it allows for high dimensional and high-throughput analysis at a relatively low cost, compared to single-cell RNA sequencing for instance.^35^ While RNA sequencing provides insights into gene expression, spectral flow cytometry offers direct information at the protein level and can analyze millions of cells, facilitating the identification of rare cell populations that might otherwise be overlooked.^35^ Nevertheless, flow cytometry loses spatial information, as samples are processed into a single-cell suspension. Combining spectral flow cytometry with advanced techniques such as high-dimensional multiplex imaging,^36^ could enhance the understanding of the spatial distribution of immune cells within both biopsy and CUSA-derived tissue fragments.

The use of the CUSA for tissue collection could potentially lead to mechanical trauma due to oscillated tissue fragmentation,^22^ which could theoretically reduce cell viability. However, brain tumors (glial or embryonal) typically exhibit a soft texture, allowing for the use of low-frequency CUSA with gentle rinsing and aspiration, limiting the risk of mechanical damage to the cells.^37^ Additionally, previous studies demonstrate that CUSA-derived tissue can be used for tumor cell culture, diagnostic purposes, and immunological research.^19–21,24^ Our findings are also consistent with earlier studies, indicating that CUSA-derived tissue may be utilized for immune profiling and T-cell culture.

CUSA-derived tissue fragments could also contain normal surrounding tissue, as CUSA is often used at the brain-tumor interface.^19^ However, our data demonstrate that despite some differences in cellular composition, the protein expression profiles of immune cells in both biopsy, and CUSA-derived tissue are comparable.

When comparing biopsy and CUSA-derived tissue, our flow cytometry analysis revealed minor differences in cellular composition. We hypothesized that the location of which a tumor biopsy is taken, such as near a blood vessel or within the tumor core, can affect the immune cell composition. For example, areas near blood vessels tend to have more lymphocytes and BMDM,^33^ while the tumor core in aggressive brain tumors often contains more neutrophils and macrophages.^34^ Neutrophil content was significantly different between biopsy and CUSA tissue fragments, where generally higher frequencies of neutrophils were observed in the CUSA-derived material. This discrepancy might be explained by a possible contamination of the CUSA tissue fragments with blood from surgery, but our analyses demonstrate that the cellular composition and protein expression profiles of immune cells found in CUSA tissue fragments are highly comparable to biopsy and significantly distinct from the peripheral blood. This suggests that immune cells in CUSA tissue fragments originate from the TIME rather than being contaminants from peripheral blood. Moreover, neutrophils have been found to infiltrate the tumor microenvironment in gliomas and brain metastases in high numbers.^38^ Therefore, the observed differences between both material types could merely be due to the differences in sample size and origin of the biopsy. This further underlines the importance of using representative samples of tumor heterogeneity for immune profiling.

We show that cryopreservation alters the cellular composition of certain cell subsets, leading to the loss of terminally differentiated cells, such as granulocytes, DCs, and resident microglia, as previously described in other studies.^29^ These differences may be related to increased sensitivity to the stresses induced by cryopreservation, such as cold stress or changes in cellular homeostasis, resulting in a reduced recovery of specific cell populations. Additionally, Granzyme B- producing cytotoxic T-cells were also found to be reduced after cryopreservation, which contrasts other studies, reporting either no change or increased frequencies of Granzyme B^+^ T-cells.^29^ On the other hand, the frequency of PD-1^+^ expressing CD4^+^ T-cells increased post- cryopreservation. While these differences in cellular composition and protein expression are fairly sudden, they should be taken into account in functional evaluations. When possible, freshly obtained samples should be preferred. However, this might be challenging, thus comparisons between fresh and cryopreserved samples should be avoided. Nevertheless, these changes were consistent across both biopsy and CUSA samples, further supporting the equivalence of these two tissue sources for immune profiling.

Interestingly, our study provided insight into the immune response within the TME, particularly the dominance of CD8^+^ T-cells, which suggests an adaptive immune response toward the tumor. This is consistent with observations in other solid tumors.^39,40^ Interestingly, CD8^+^ T-cells in the brain TIME retained their cytotoxic potential, as evidenced by their granzyme B expression. This contrasts with other solid tumors where T-cells exhibit an exhausted phenotype due to the immune suppressive environment and chronic antigen exposure.^41–43^ In line with our data, Upadhye et al. demonstrated that elevated PD1-expression on intra-tumoral T-cells did not indicate exhaustion, but rather signified clonal expansion and tumor reactivity.^40^ Furthermore, our data on T-cell proliferation and cytokine/cytolysin production upon stimulation suggest that CD8^+^ T cells retain their functionality when removed from the suppressive brain tumor environment.^30^ Together, these findings demonstrate the potential of utilizing CUSA tissue fragments as a source for the development of immunotherapeutic approaches, such as TIL or CAR-therapy.

Finally, we highlight the importance of separating CUSA tissue fragments from the liquid fraction, as immune cells from the liquid fraction display a phenotype that is intermediate between in the TIME and peripheral blood, while tumor cells, though fewer in number, are still present. This suggests that the liquid fraction could be useful for tumor cell phenotyping and culture, further enhancing the utility of CUSA-derived materials for both immune and oncological research.^19^

As our study was performed on a relatively small number of patients, future studies should focus on validating these findings across a larger cohort of patients and more diverse tumor types, as well as exploring the potential of CUSA-derived samples for advanced molecular analyses such as single-cell sequencing and spatial transcriptomics. Additionally, investigating the utility of CUSA-derived T-cells in different functional assays, such as immune checkpoint blockade, could provide valuable insights into treatment response, potentially paving the way for more effective immunotherapeutic strategies in high-grade brain tumors.

In conclusion, this study demonstrates that CUSA-derived tissue fragments are a valuable resource for the immunophenotype of the TIME in pediatric brain tumors, offering a viable alternative to biopsy material. Furthermore, our preliminary functional analyses provide evidence on the functional potential of CUSA-derived T-cells, opening new avenues for immunotherapies. Therefore, leveraging CUSA-derived material for immune-oncological research might deepen our understanding of the brain TIME.

## Supplementary Material

vdaf097_suppl_Supplementary_Tables_S1-S3_Figures_S1-S8

## Data Availability

Main data produced in this study are presented within the article. The authors will provide access to raw data and materials upon reasonable request.
