# Supplementary material for: Profiling the immune tumor microenvironment of pediatric brain tumors with cavitron ultrasonic surgical aspirator (CUSA)-derived tissue fragments
Source: Neurooncol Adv. 2025 May 17;7(1):vdaf097. doi: 10.1093/noajnl/vdaf097 (PMC12448718; doi:10.1093/noajnl/vdaf097)
Supplement: vdaf097_suppl_Supplementary_Tables_S1-S3_Figures_S1-S8 [file vdaf097_suppl_supplementary_tables_s1-s3_figures_s1-s8.docx]

**Profiling the immune tumor microenvironment of pediatric brain tumors with Cavitron Ultrasonic Surgical Aspirator (CUSA) derived tissue fragments**

Joyce Meesters-Ensing^1^, Mariëtte E.G. Kranendonk^1^, Raoul Hoogendijk^1^, Eelco Hoving^1^, Friso G. Calkoen^1^, Jasper van der Lugt^1^, Tiago Carvalheiro^1¥^, Stefan Nierkens^1,2¥^*

^1^Princess Máxima Center for Pediatric Oncology, Utrecht, the Netherlands;

^2^Center for Translational Immunology, University Medical Center Utrecht, Utrecht University, Utrecht, The Netherlands.

^¥^ These authors contributed equally as last authors

***Corresponding author:**

Stefan Nierkens, PhD

Princess Máxima Center for Pediatric Oncology

Heidelberglaan 25

3584 CS Utrecht

The Netherlands

Tel: +31 (0) 88 972 7272

S.Nierkens-2@prinsesmaximacentrum.nl

**Supplementary figures and tables**


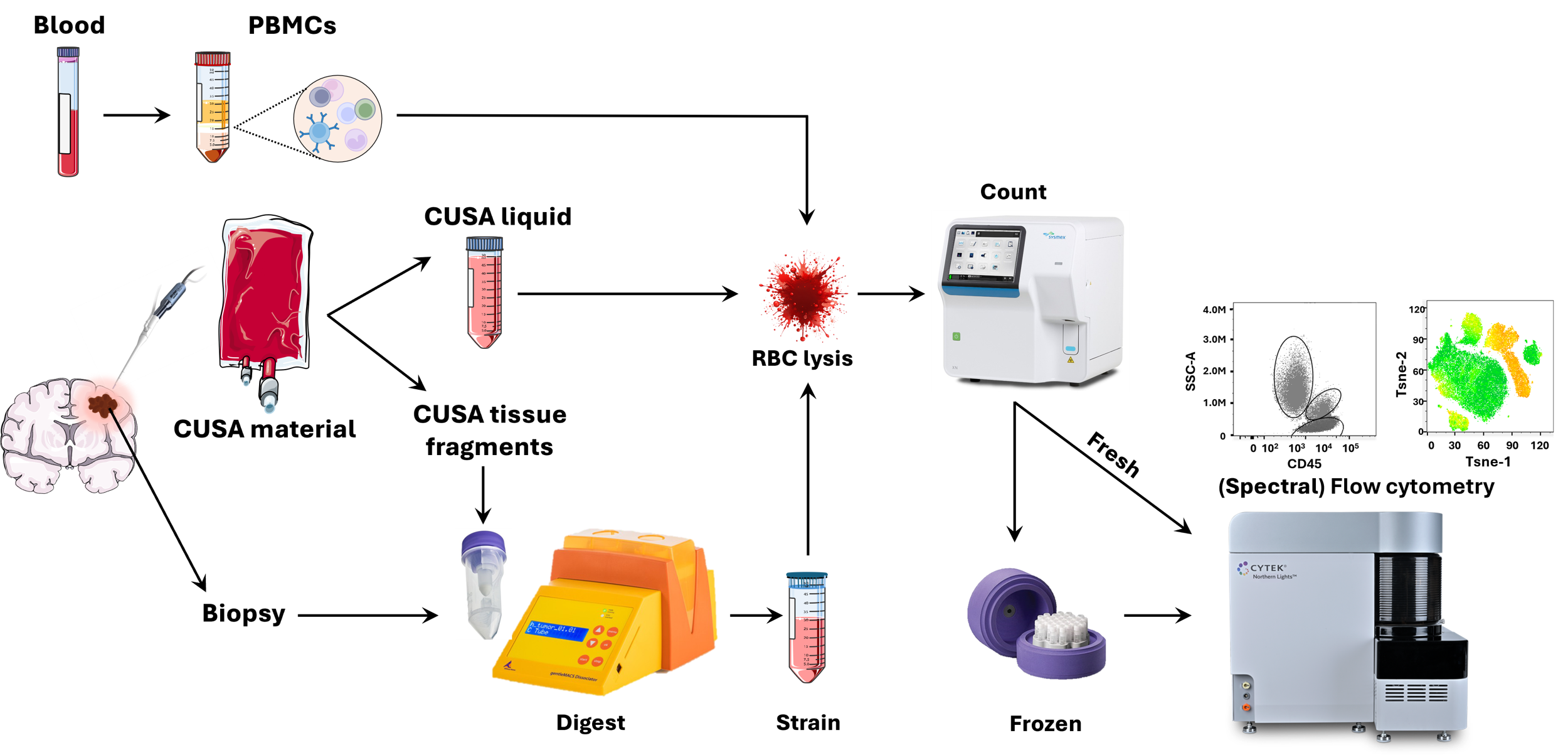


**Supplementary figure 1: Sample processing overview.** Peripheral blood mononuclear cells (PBMCs) were isolated from whole blood by density gradient centrifugation. CUSA-derived material was filtered to separate tissue fragments and the liquid fraction. Biopsy and CUSA tissue fragments were digested and strained, followed by red blood cell (RBC) lysis. The amount of white blood cells was estimated with Sysmex cell counter and samples were either freshly used or cryopreserved and then analyzed using spectral flow cytometry. Some graphical elements in this figure were adapted from Servier Medical Art (smart.servier.com), licensed under CC BY 4.0.


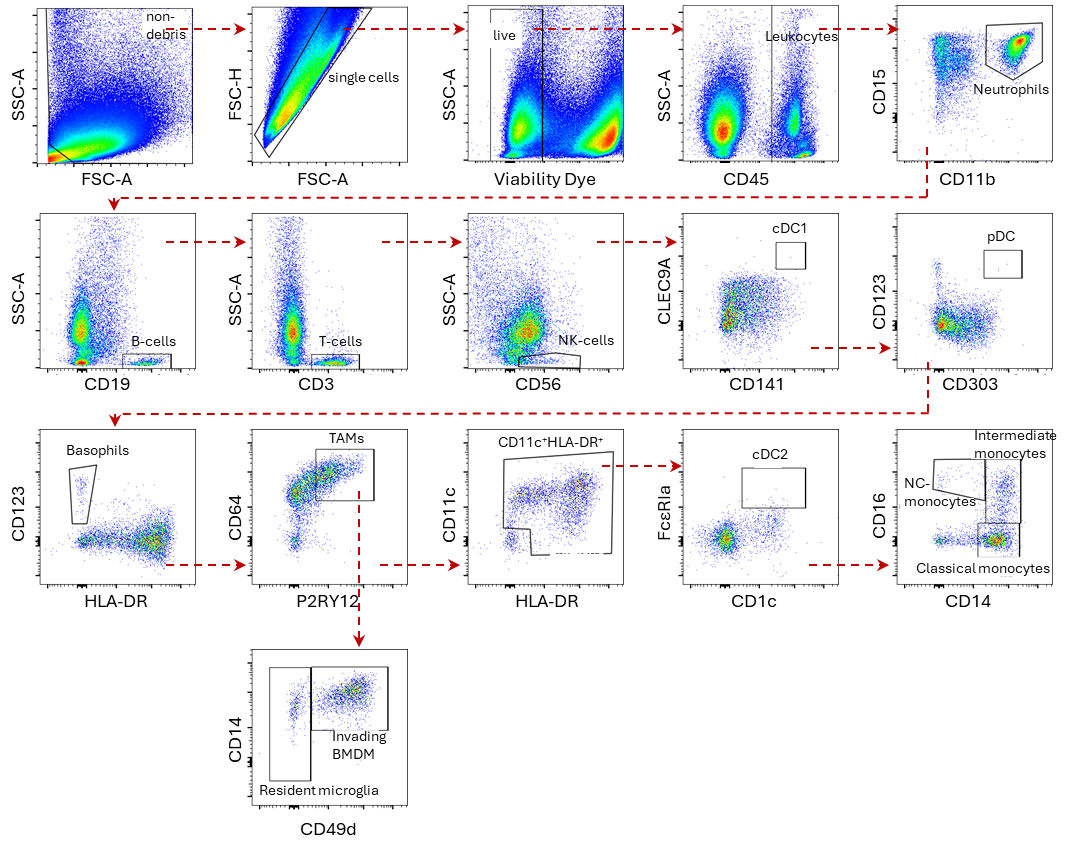


**Supplementary figure 2: Flow cytometry gating strategy to identify the different myeloid cell populations.** Analysis strategy includes exclusion of cell debris, doublets and dead cells before identifying the CD45^+^ leukocyte population. The different immune cell types were identified: neutrophils (CD11b^+^CD15^+^), B-cells (CD19^+^SSC^low^), T-cells (CD3^+^SSC^low^), natural killer (NK) cells (CD56^+^SSC^low^), cDC1 (CD141^+^CLEC9A^+^), pDC (CD123^+^CD303^+^), Basophils (CD123^+^HLA-DR^+^), tumor associated macrophages (TAMs) (CD64^+^P2RY12^+^), cDC2 (CD1c^+^CD11c^+^FcεRIa^+^HLA-DR^+^), Classical monocytes (CD14^+^CD16^-^), Intermediate monocytes (CD14^+^CD16^+^), Non-classical monocytes (CD14^-^CD16^+^). TAMs were subdivided into resident microglia (CD49d^-^) and invading bone marrow derived macrophages (BMDM) (CD49d^+^). cDC = conventional dendritic cell; pDC = plasmacytoid dendritic cell.

**
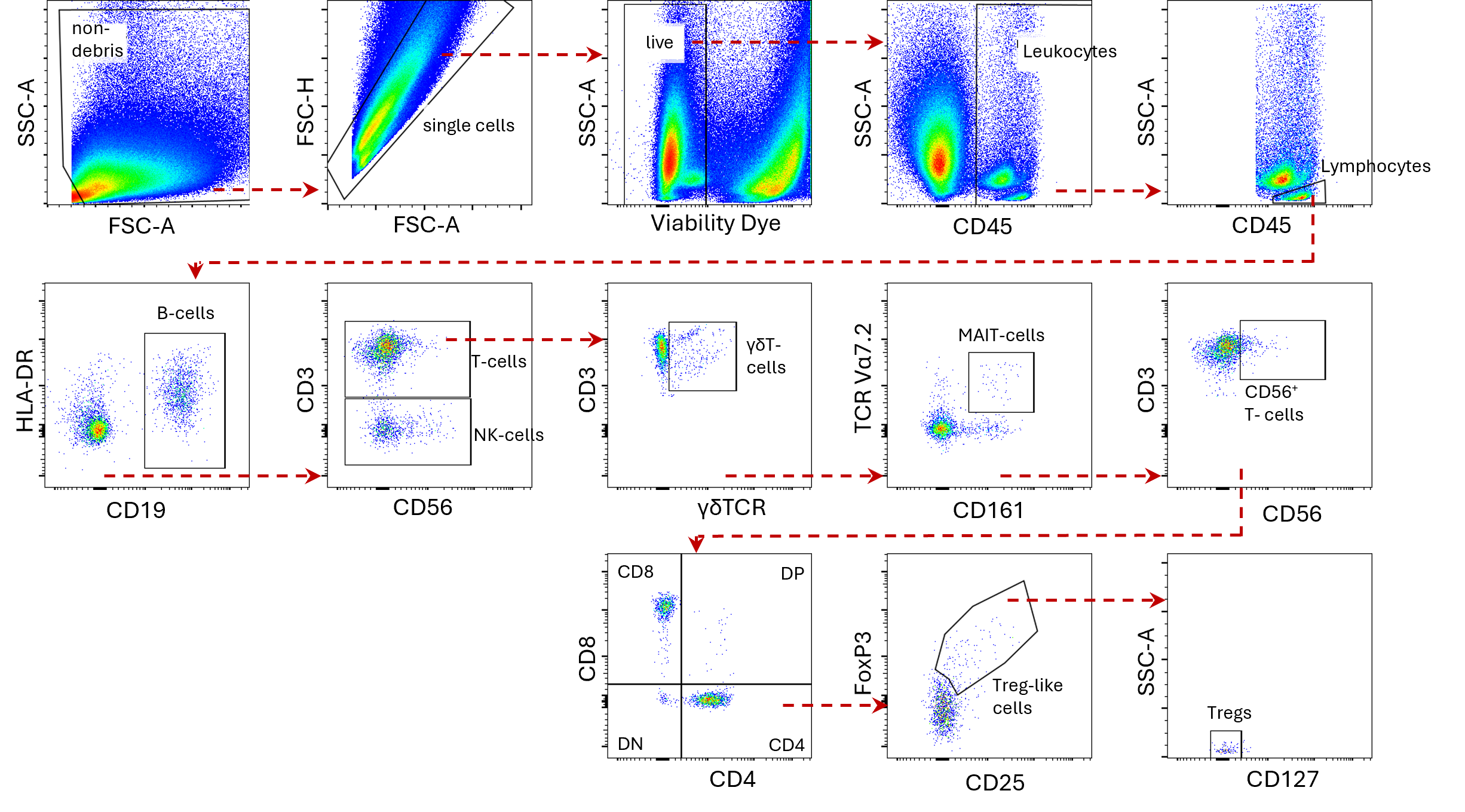
Supplementary figure 3: Flow cytometry gating strategy to identify the different lymphoid cell populations.** Analysis strategy includes exclusion of cell debris, doublets and dead cells before identifying the CD45^+^ leukocyte population and subsequent lymphocytes (CD45^high^SSC^low^). The different lymphoid populations were identified as: B-cells (CD19^+^HLA-DR^+^), T-cells (CD3^+^), natural killer (NK) cells (CD56^+^), gamma-delta (γδ) T-cells (CD3^+^γδTCR^+^), Mucosal associated invariant T (MAIT)-cells (CD161^+^TCRVα7.2^+^), CD56^+^ T-cells (CD3^+^CD56^+^), CD4^+^ T-cells (CD4^+^CD8^-^), double positive (DP) T-cells (CD4^+^CD8^+^), CD8^+^ T-cells (CD4^-^CD8^+^), double negative (DN) T-cells (CD4^-^CD8^-^) and regulatory T-cells (T_regs_) (CD4^+^CD25^+^CD127^-^FoxP3^+^). NK cells were subdivided into immature (CD56^+^CD16^-^), intermediate (CD56^-^CD16^-^)and mature NK-cells (CD56^-^CD16^+^). γδT-cells were subdivided into Vδ1^+^ and Vδ2^+^.

**
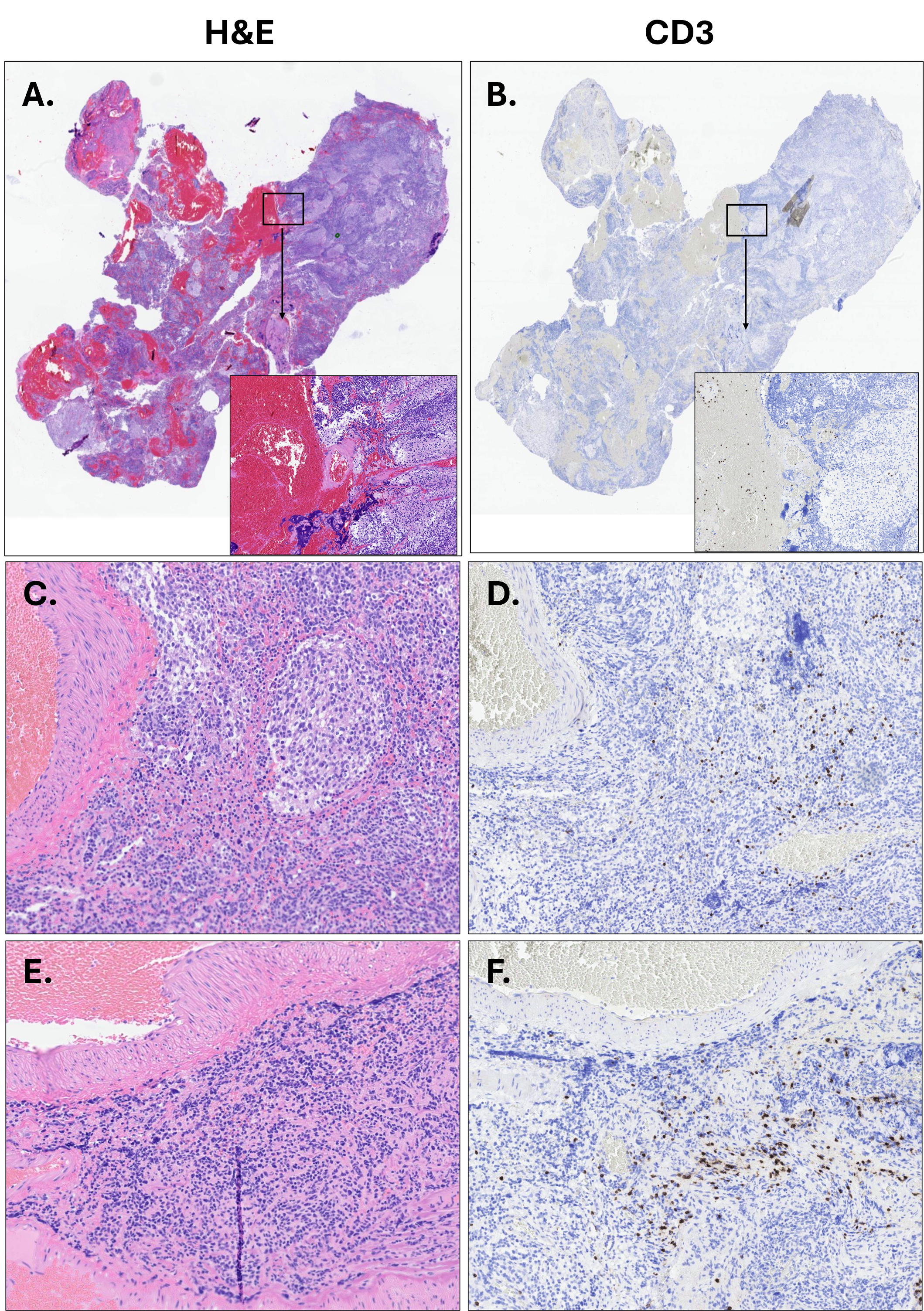
**

**Supplementary figure 4: T-cell infiltration distribution in tumor biopsy samples. A, C and E)** Hematoxylin and eosin (H&E) staining showing cell nuclei (purple), extracellular matrix and cytoplasm (pink), and red blood cells (red). **B, D and F)** Immune histochemistry (IHC) staining for CD3 shows T-cells in brown. Representative images of n=2 medulloblastoma patient samples with the highest T-cell content as determined by flow cytometry (related with Fig. 1C). **A, B)** Images from Patient 1: overview (original scale) and zoomed region (bottom right; ×100 magnification); **(C–F)** Images from Patient 2, all at ×100 magnification.


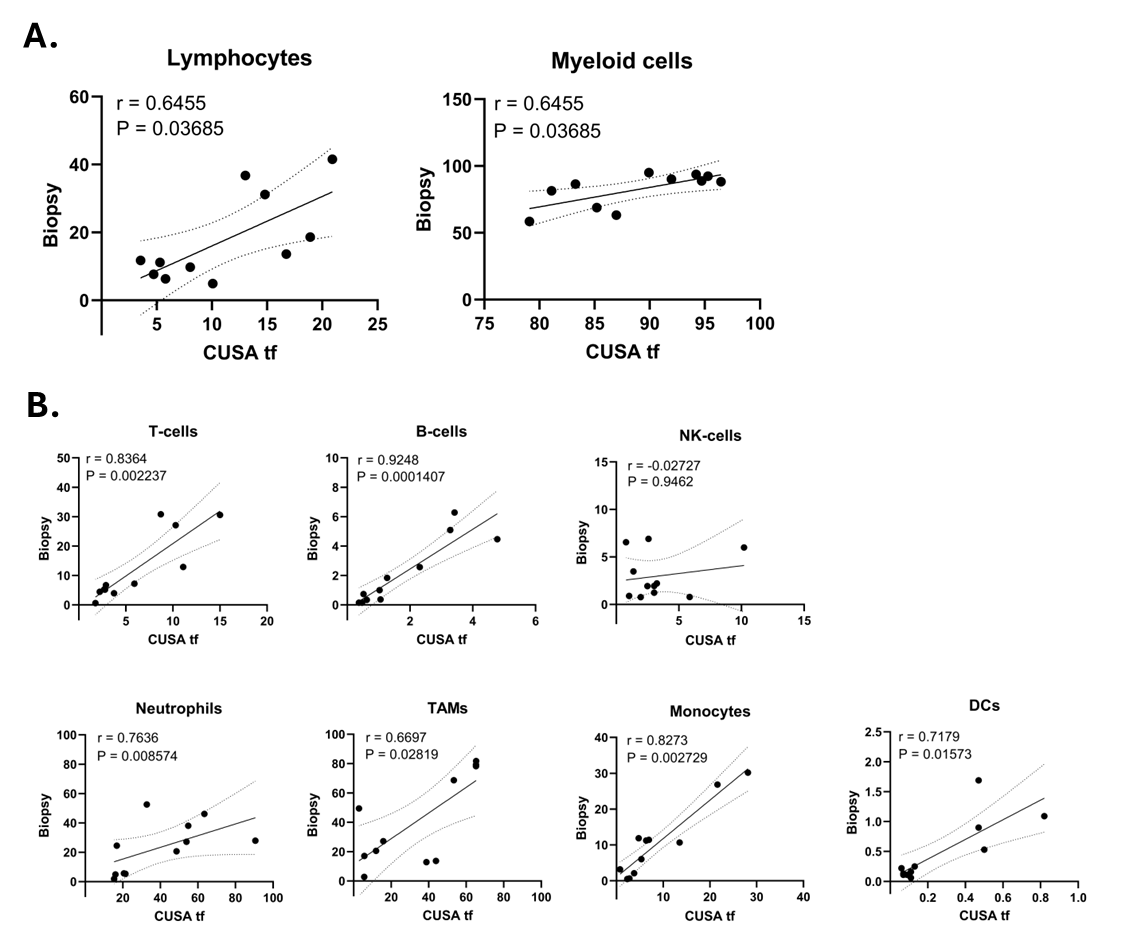


**Supplementary figure 5: Correlation of immune cell subset frequencies between freshly isolated biopsy and CUSA tissue fragments.** Correlations between biopsy and CUSA tissue fragments (tf) for **A)** lymphoid and myeloid cells and **B)** lymphoid (T-, B-, NK-cells) and myeloid (neutrophils, TAMs, monocytes and DCs) subsets . Analysis was performed without normalization for neutrophil content. TAM = tumor associated macrophage; DC = dendritic cell. Correlations were calculated using a non-parametric Spearman test; n=11.

**
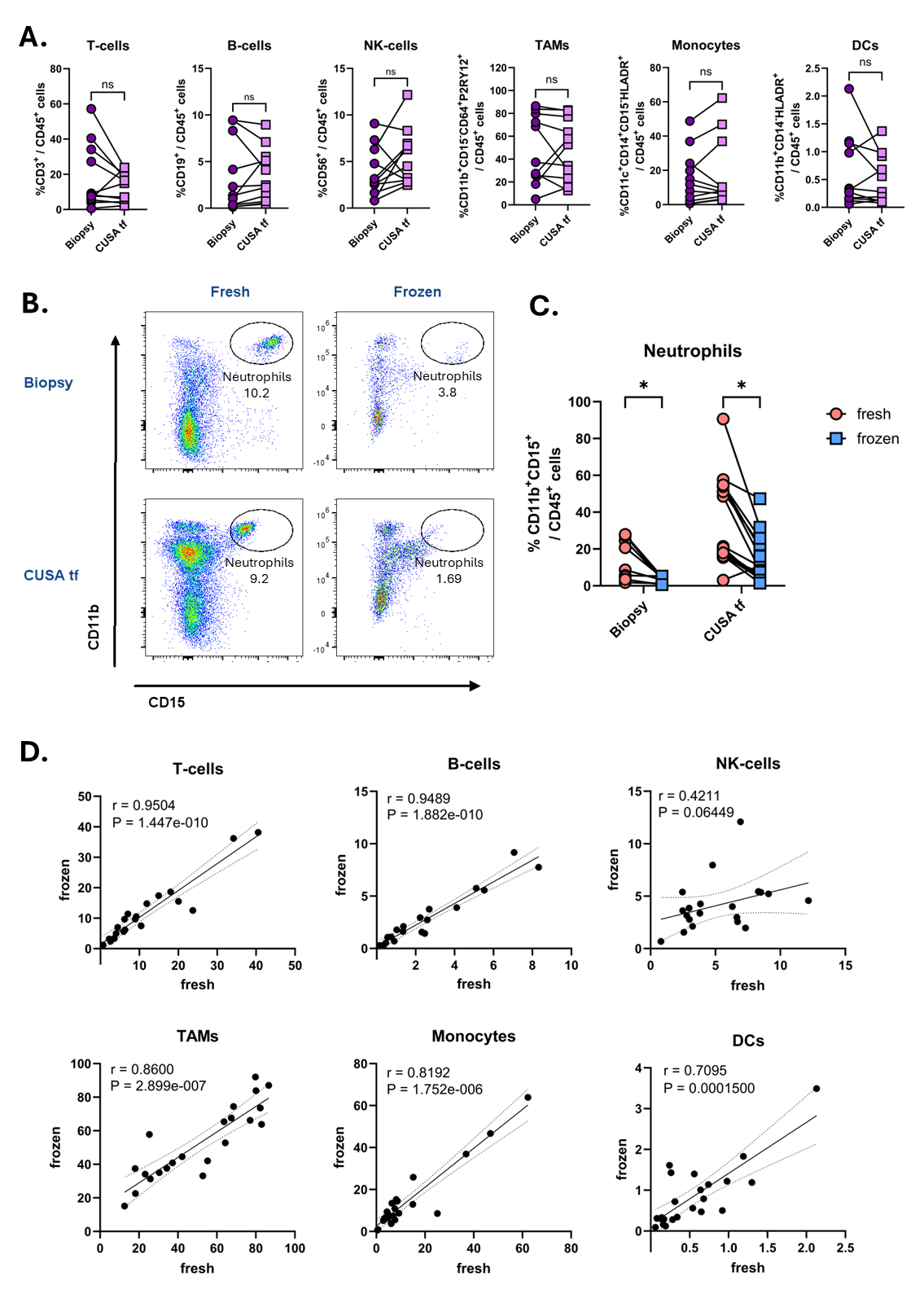


Supplementary figure 6: Impact of the neutrophil presence on immune cell population quantification.** **A)** Quantification of the different immune cell populations from freshly isolated biopsy and CUSA tissue fragments (tf), excluding neutrophil contribution to the CD45^+^ population; n=11. **B)** Flow cytometry plots representing the gating strategy for neutrophils (CD45^+^CD11b^+^CD15^+^) present in biopsy and CUSA tissue fragment material, in both freshly isolated and cryopreserved samples. **C)** Quantification of the percentage of neutrophils after cryopreservation in matched biopsy and CUSA tissue fragment samples; n= 8 biopsy, n=15 CUSA tf. **D)** Correlation for the different immune cell populations in fresh and cryopreserved samples, excluding neutrophils; n=8 biopsy, n=15 CUSA tf. TAM = tumor associated macrophage; DC = dendritic cell. Statistical differences in a Wilcoxon paired t-test are depicted as ns = non significant and * = p<0.05. Correlations were calculated using a non-parametric Spearman test.


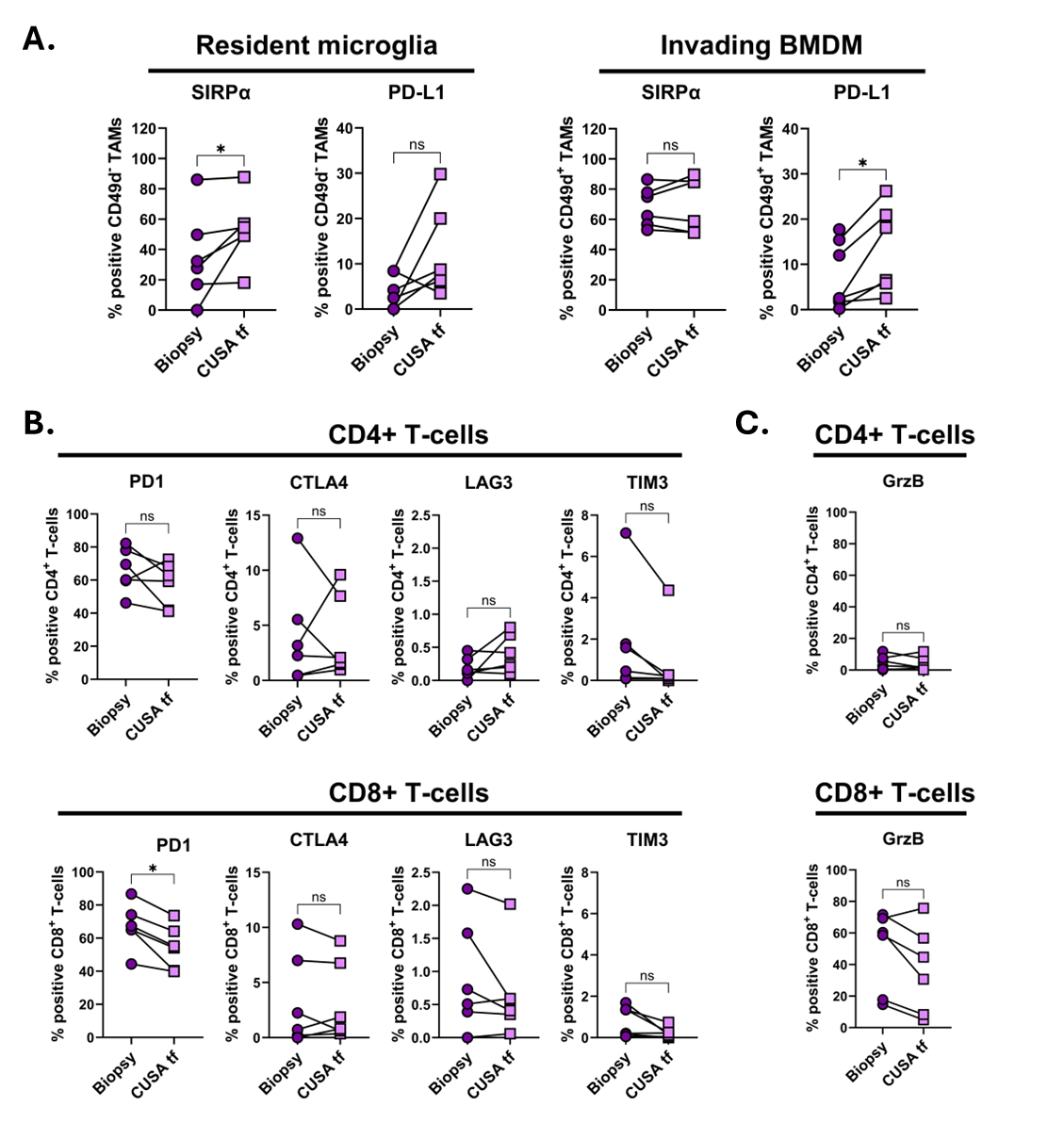


**Supplementary figure 7: Protein expression profiles on biopsy and CUSA tissue fragments after cryopreservation.** **A)** Protein expression levels of SIRPα and PD-L1 on both resident microglia and invading bone marrow derived macrophages (BMDM) in biopsy and CUSA tissue fragments (tf) after cryopreservation. **B)** PD1, CTLA4, LAG3 and TIM3 checkpoint receptor expression levels on CD4^+^ and CD8^+^ T-cells in the different matched types of samples after cryopreservation. **C)** Granzyme B (GrzB) production by both CD4^+^ and CD8^+^ T-cells after cryopreservation. Statistical differences in a Wilcoxon paired t-test are depicted as ns = non significant and * = p<0.05; n=6.

**
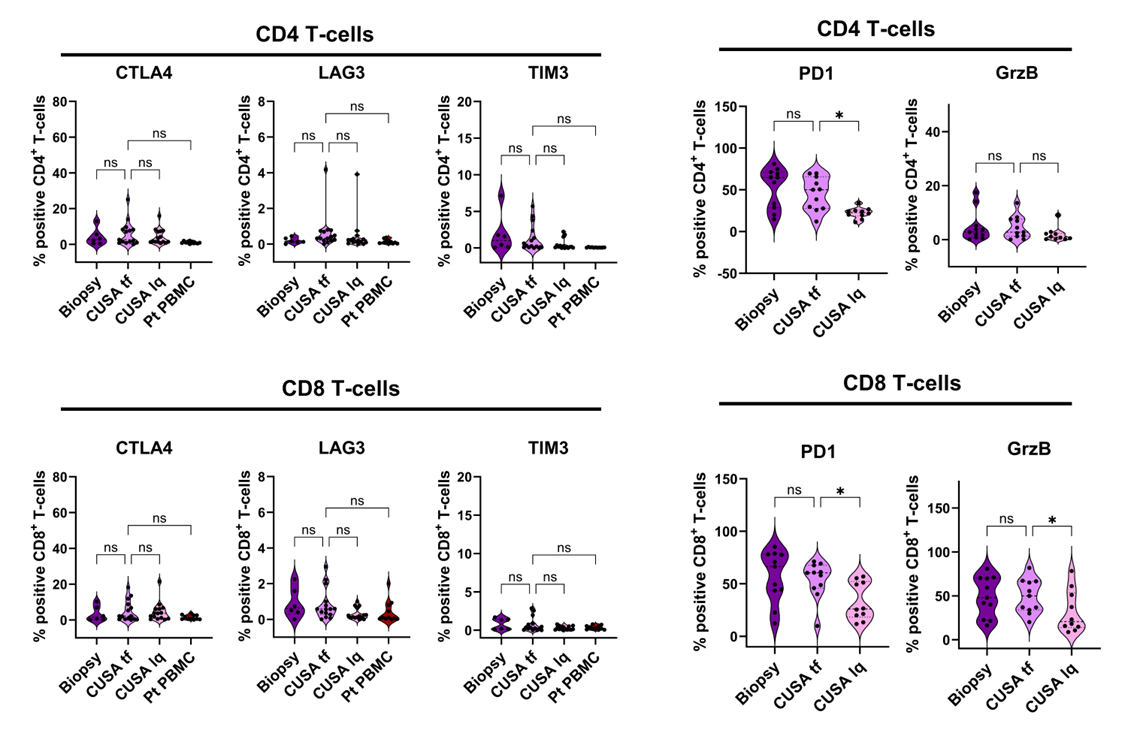
Supplementary figure 8: Protein expression profiles on T-cells in the TIME are significantly different compared to the peripheral blood and CUSA liquid fraction.** **A)** CTLA4, LAG3 and TIM3 checkpoint receptor expression levels on CD4^+^ and CD8^+^ T-cells in the TIME (biopsy and CUSA tissue fragments (tf)), CUSA liquid fraction and peripheral blood of matched patient (Pt) samples after cryopreservation; n=6 Biopsy, n=15 CUSA tf, n=15 CUSA lq, n=11 Pt PBMC. **B)** PD1 and Granzyme B (GrzB) expression on both CD4^+^ and CD8^+^ T-cells in the TIME compared to the CUSA liquid fraction of fresh samples; n=11. Statistical differences using a mixed effect model with Greisser-Greenhouse correction and only matched samples are depicted as ns = non-significant, * = p<0.05 and ** = p<0.01.


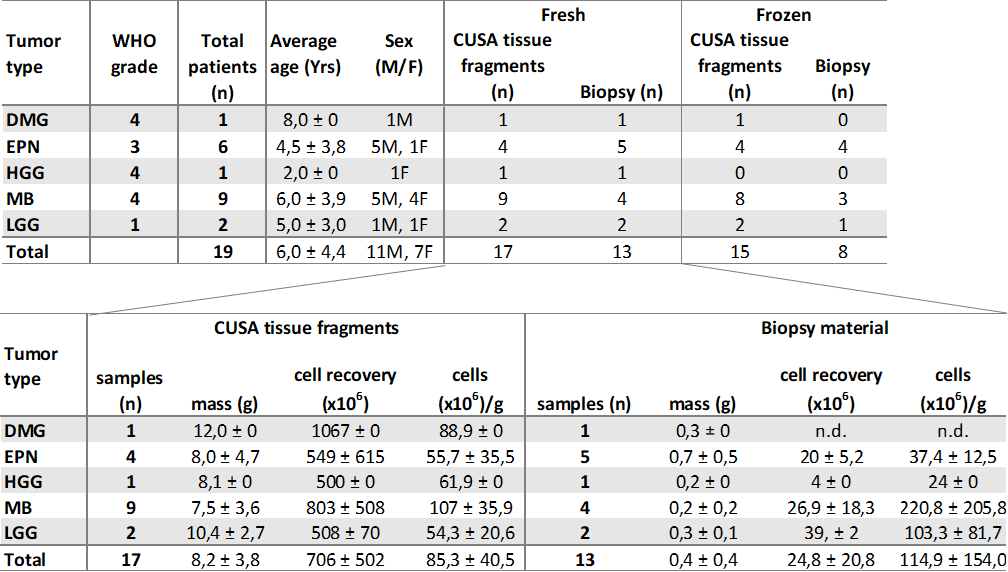


**Supplementary table 1: Patient and sample characteristics.** All samples were obtained during surgery after first diagnosis. DMG = diffuse midline glioma; EPN = ependymoma; HGG = high grade glioma; MB = medulloblastoma; LGG = low grade glioma.

| **#** | **Fluorophore** | **Marker** | **Supplier** | **Catalog no.** |
| --- | --- | --- | --- | --- |
| **1** | BUV496 | CD11b | BD | 750065 |
| **2** | BUV615 | CD49d | BD | 751458 |
| **3** | BUV805 | CD16 | BD | 748850 |
| **4** | BV421 | CD1c | Biolegend | 331526 |
| **5** | Pacific Blue | CD45 | Biolegend | 304029 |
| **6** | BV480 | PD-L1 | BD | 746346 |
| **7** | cFluor V547 | HLA-DR | Biolegend | 307638 |
| **8** | BV711 | CD172A/B | BD | 743566 |
| **9** | FITC | CD56 | Biolegend | 318304 |
| **10** | cFluor B548 | CD15 | Cytek | R7-20025 |
| **11** | PerCP | CD3 | Biolegend | 300427 |
| **12** | PerCP-Cy5.5 | CD11c | Biolegend | 371518 |
| **13** | PerCP-eFluor 710 | FcεRIa | Thermo Fisher | 46-5899-42 |
| **14** | PE | CLEC9A | Biolegend | 353804 |
| **15** | PE/Dazzle549 | P2RY12 | Biolegend | 392111 |
| **16** | PE-Cy5 | TIM-3 | Biolegend | 345052 |
| **17** | cFluor BYG710 | CD19 | Cytek | R7-20009 |
| **18** | PE-Cy7 | CD303 | Biolegend | 354214 |
| **19** | APC | CD141 | Biolegend | 344105 |
| **20** | Alexa Fluor 700 | CD123 | Biolegend | 306040 |
| **21** | APC-Fire 750 | CD64 | Biolegend | 305035 |
| **22** | ViaDye Red | ViaDye Red | Cytek | R7-60008 |
| **23** | APC-Fire 810 | CD14 | Biolegend | 367156 |

**Supplementary table 2: Myeloid panel antibodies**

| **#** | **Fluorophore** | **Marker** | **Supplier** | **Catalog no.** |
| --- | --- | --- | --- | --- |
| **1** | BUV496 | CD3 | BD | 612940 |
| **2** | BUV615 | Vδ2 | BD | 751368 |
| **3** | BUV661 | ydTCR | BD | 750019 |
| **4** | BUV805 | CD4 | BD | 742000 |
| **5** | BV421 | CD161 | Biolegend | 339914 |
| **6** | Vioblue | Vδ1 | Miltenyi | 130-120-443 |
| **7** | cFluor V547 | CD45 | Cytek | R7-20011 |
| **8** | BV605 | CD25 | Biolegend | 302632 |
| **9** | BV750 | PD-1 | Biolegend | 329966 |
| **10** | BV785 | Vα7.2 TCR | Biolegend | 351722 |
| **11** | Alexa Fluor 488 | FoxP3 | Biolegend | 320112 |
| **12** | cFluor B548 | CD14 | Cytek | R7-20115 |
| **13** | PerCP | HLA-DR | Tonbo-Bio | 67-9952-T100 |
| **14** | PerCP-Cy5.5 | GrzB | Biolegend | 372212 |
| **15** | PE | CTLA4 | Biolegend | 369604 |
| **16** | PE-Cy5 | TIM-3 | Biolegend | 345052 |
| **17** | cFluor BYG710 | CD19 | Cytek | R7-20009 |
| **18** | PE-Cy7 | LAG-3 | Biolegend | 369310 |
| **19** | cFluor R668 | CD16 | Cytek | R7-20069 |
| **20** | cFluor R720 | CD127 | Cytek | RC-00004 |
| **21** | APC-Fire 750 | CD56 | Biolegend | 392408 |
| **22** | ViaDye Red | Live/dead | Cytek | R7-60008 |
| **23** | APC-Fire 810 | CD8 | Biolegend | 344764 |

**Supplementary table 3: TBNK panel antibodies**
